# Frequency and correlates of inaccurate psychiatric diagnoses in 401 adults hospitalized for major depressive disorder: a comparison of clinical versus research interview-based diagnoses

**DOI:** 10.1017/S109285292610100X

**Published:** 2026-06-09

**Authors:** Viktor Benjamin Nöhles, Till Julius Adam, Felix Bermpohl, Nikola Schoofs, Rouven Bathe-Peters, Nina Hilpert, Forugh Salimi Dafsari, Peter Falkai, Cornelius Schüle, Elena Wang, Christine Reif-Leonhard, Andreas Reif, Susanne Schillo, Rabia Sana Arshad, Patricia Getty, Christian Otte, Dominique Piber, Livia Graumann, Sebastian Weyn-Banningh, Mazda Adli, René Papenfuß, Andreas Meyer-Lindenberg, Stefan Fritze, Michael Bauer, Ute Lewitzka, Maik Spreer, Clemens Karl Krug, Kerstin Rubarth, Christoph Ulrich Correll

**Affiliations:** 1 Department of Child and Adolescent Psychiatry, Psychosomatic Medicine and Psychotherapy, Charité – Universitätsmedizin Berlin, corporate member of Freie Universität Berlin and Humboldt-Universität zu Berlin, Campus Virchow-Klinikum, Berlin, Germany; 2 German Center for Mental Health (DZPG), partner site Berlin, Berlin, Germany; 3 Department of Psychiatry and Neurosciences, Charité – Universitätsmedizin Berlin, corporate member of Freie Universität Berlin and Humboldt-Universität zu Berlin, Berlin, Germany; 4 Department of Psychiatry and Psychotherapy, Faculty of Medicine and University Hospital Cologne, University of Cologne, Cologne, Germany; 5 Clinic for Psychiatry and Psychotherapy, Ludwig Maximilian University of Munich, Munich, Germany; 6 German Center for Mental Health (DZPG), partner site Munich, Munich, Germany; 7 Max Planck Institute for Psychiatry, Munich, Germany; 8 Department of Psychiatry, Psychosomatic Medicine and Psychotherapy, University Medical Center Frankfurt, Frankfurt am Main, Germany; 9 Fraunhofer Institute for Translational Medicine and Pharmacology ITMP, Frankfurt am Main, Germany; 10 Department of Psychiatry and Psychotherapy, Charité – Universitätsmedizin Berlin, corporate member of Freie Universität Berlin and Humboldt-Universität zu Berlin, Campus Benjamin Franklin, Berlin, Germany; 11 Department of Psychiatry and Neurosciences, Charité – Universitätsmedizin Berlin, corporate member of Freie Universität Berlin and Humboldt-Universität zu Berlin, Campus Charité Mitte, Berlin, Germany; 12 Center for Psychiatry, Psychotherapy and Psychosomatic Medicine, Fliedner Klinik Berlin, Berlin, Germany; 13 Department of Psychiatry and Psychotherapy, Central Institute of Mental Health, Mannheim, Germany; 14 German Center for Mental Health (DZPG), partner site Mannheim, Mannheim, Germany; 15 Department of Psychiatry and Psychotherapy, University Hospital Carl Gustav Carus, Faculty of Medicine, Technische Universität Dresden, Dresden, Germany; 16 Institute of Biometry and Clinical Epidemiology, Charité – Universitätsmedizin Berlin, corporate member of Freie Universität Berlin and Humboldt-Universität zu Berlin, Berlin, Germany; 17 The Zucker Hillside Hospital, Psychiatry Research, Northwell Health, Glen Oaks, NY, USA; 18 Department of Psychiatry and Molecular Medicine, Zucker School of Medicine at Hofstra/Northwell, Hempstead, NY, USA

**Keywords:** Depression, discordance, diagnosis, inpatient, research interview, bipolar disorder, substance use disorder

## Abstract

**Objective:**

To evaluate the validity and correlates of inaccurate clinically diagnosed moderate/severe ICD-10 major depressive disorder (MDD) and psychiatric comorbidities in adult inpatients.

**Methods:**

The multicenter Patient Characteristics, Validity of Clinical Diagnoses and Outcomes Associated with Suicidality in Inpatients with Symptoms of Depression (OASIS-D) study enrolled 18- to 75-year-old inpatients with MDD who underwent assessments for clinical, illness, and treatment characteristics, including the Mini-International-Neuropsychiatric-Interview (MINI). False-positive clinically MDD diagnoses were characterized via MINI. Diagnostic agreement between clinical and MINI-based psychiatric comorbidities was assessed with kappa-statistic.

**Results:**

Among 401 patients (median age = 33.0 years; female = 57.1%), 42 (10.5%) were false positive for MDD (i.e., different primary diagnosis). Reasons for misdiagnosing MDD exclusive to false-positive cases included bipolar disorder (45.2%), subsyndromal depression (16.7%), and physical illness-related/substance use disorder (SUD)-related depression (4.8%), whereas missed diagnoses that could also occur in true-positive, MINI-based MDD patients included primary SUDs (23.8%) and primary anxiety disorders (9.5%). Among comorbidities, greatest disagreement between clinical and MINI-based research diagnoses existed for antisocial personality disorder (0% vs. 5.2%, *κ* =0), followed by anxiety disorders (7.5% vs. 45.1%, *κ* =0.17), obsessive-compulsive disorder (3.0% vs. 8.7%, *κ* =0.27), SUDs (11.0% vs. 28.4%, *κ* =0.29), eating disorders (3.5% vs. 7.2%, *κ* =0.39), and posttraumatic stress disorder (10.7% vs. 23.7%, *κ* =0.40).

**Conclusions:**

Bipolar depression and SUDs contributed to two thirds of misdiagnosed MDD. Missed comorbidities included mostly anxiety disorders, SUDs, and PTSD. Accurate diagnostic assessment, including longitudinal history, is essential to reduce misdiagnosis and ensure guideline-based treatment for psychiatric disorders in MDD inpatients.

## Introduction

Major depressive disorder (MDD) is one of the most common mental disorders worldwide, with a global point prevalence of 4.7%^1^ and a lifetime prevalence of >10%.^2^ ^,^^3^ In 2015, MDD affected >200 million people worldwide and was the second leading cause of years lived with disability.^4^ Individuals with MDD frequently experience functional impairment, including absenteeism, diminished work productivity, and impaired daily activity.^5^ Moreover, MDD is associated with other psychiatric and somatic comorbidities,^6^ ^,^^7^ leading to diminished quality of life^8^ and premature mortality.^6^ ^,^^9^ The economic burden of MDD is substantial—in 2012, the annual economic burden of MDD across Europe was approximately €91.9 billion ($102.9 billion), with direct health care and nonmedical costs of €37.8 billion ($42.3 billion) and indirect costs of €53.9 billion ($60.4 billion).^10^

To provide the most effective support to patients with MDD and reduce the healthcare system burden, it is essential to implement evidence-based interventions. However, misdiagnosis can lead to inappropriate treatment and suboptimal outcomes. For instance, misclassifying bipolar disorder (BD) as MDD can result in antidepressant monotherapy rather than mood-stabilizing treatment, increasing the risk of mania, raising both symptom burden and healthcare costs.^11^ ^,^^12^

Therefore, achieving optimal treatment necessitates accurate diagnoses. However, clinical diagnoses often differ from those derived from the systematic application of formal criteria. Transdiagnostically, the level of agreement between clinical and research-based psychiatric diagnoses has been reported as fair ( *κ* =0.27) in a meta-analysis of 38 studies involving 15 967 adults in mixed in-/outpatient and primary care/psychiatric settings^13^ and as moderate ( *κ* =0.48) in another meta-analysis of 13 studies (*n* = 9854) conducted in in-/outpatient psychiatric settings, including adolescents and adults.^14^

For example, a meta-analysis of 19 studies of general practitioners estimated that 58% of MDD diagnoses were discordant (ie, false positive or false negative), assuming an urban prevalence rate for MDD of 20%.^15^ Although somewhat lower than in primary care, studies conducted within psychiatric services found that 24.6%–55.2% of clinical MDD diagnoses were not confirmed by structured diagnostic instruments.^16^ ^–^^20^

Furthermore, predictors of discordant MDD diagnoses may sensitize clinicians to identify patients at risk of misdiagnosis. Two studies involving general practitioners or mental health professionals, conducted either solely in outpatients (*n* = 372)^21^ or in outpatient/partial hospitalization settings (*n* = 5639),^22^ revealed several significant correlates of discordance between clinical and research-based MDD diagnoses. Predictors of diagnostic discordance included higher age (35–49 years or 65+ years vs. 18–25 years) and lower educational attainment, as well as several consistent indicators of lower illness severity and better functioning, including lower depression severity, absence of suicidal ideation/attempts, less severe mental illness, no mental health treatment, being married (vs. being divorced/separated), being employed (vs. being unemployed), and having better self-rated overall health and overall functioning.^21^ ^,^^22^ Conversely, another study of 493 outpatients with MDD found no significant multivariable predictors.^16^

In addition to identifying predictors of misdiagnosed MDD, recognizing and accurately diagnosing comorbid psychiatric conditions is equally essential for achieving diagnostic precision and optimizing treatment outcomes. Comorbid disorders, like alcohol/substance use disorders (ASUDs), anxiety disorders, obsessive-compulsive disorder (OCD), and posttraumatic-stress disorder (PTSD), have been identified as predictors of nonremission and reduced treatment adherence, a more chronic course of depression, higher hospitalization rates, and an increased risk of suicidal behavior in patients with MDD.^23^ ^,^^24^ If not specifically assessed and recognized, such comorbidities may increase the overall symptom burden and reduce treatment effectiveness. Importantly, diagnostic agreement between clinical and research-based diagnoses also varies across other psychiatric disorders. Meta-analyses have reported substantial agreement for eating disorders (*κ* = 0.70)^13^ and OCD (*κ* = 0.64),^13^ followed by moderate agreement for PTSD (*κ* = 0.54),^13^ fair to moderate agreement for ASUDs (*κ* = 0.56; *κ* ≈ 0.30),^13^ ^,^^14^ and fair agreement for anxiety disorders (*κ* = 0.29; *κ* ≈ 0.35).^13^ ^,^^14^

Despite extensive research, only few studies have systematically examined the agreement between clinical and structured research diagnoses of MDD and comorbid conditions in psychiatric inpatients. Given the high clinical stakes associated with severe depressive episodes and suicidality, diagnostic accuracy is crucial to guide appropriate treatment. Therefore, the current study, using data from Patient Population-2 (PP2) of the Patient Characteristics, Validity of Clinical Diagnoses and Outcomes Associated with Suicidality in Inpatients with Symptoms of Depression (OASIS-D) study,^25^ aimed to assess the (i) accuracy of clinical versus research-based diagnoses of MDD in patients hospitalized for clinically diagnosed MDD; (ii) concordance/discordance of psychiatric comorbidities; and (iii) clinical and demographic predictors of diagnostic inaccuracy of MDD and discordance among comorbidities. We hypothesized a priori based on the prior literature^15^ ^–^^20^ that <50% of clinical MDD diagnoses would not be confirmed by a structured research interview. We further hypothesized that psychiatric comorbidities would be underdiagnosed to varying degrees. Moreover, we hypothesized a priori^25^ that an inaccurate clinical MDD diagnosis would be associated with younger age (less historical information/more dynamic emergence of [co]morbidities), female sex (diagnostic bias), lower number of previous depressive episodes (less historical validation), lower number of psychiatric hospitalizations (less historical validation), higher number of comorbid personality disorders (diagnostic overlap), and lower suicidal severity (less severe psychiatric condition). Finally, based on the literature cited above,^21^ ^,^^22^ we hypothesized that discordance in the psychiatric comorbidities would be associated with milder illness severity and better functioning.

## Methods

### Observational design

The present study focuses on the second of three patient populations of the multicenter OASIS-D study in adults ≥18–75 years old consecutively hospitalized for MDD (International Classification of Diseases, 10th Revision [ICD-10]^18^: F32, F33) and with at least passive suicidal thoughts present ≥48 hours after admission (the study design has been reported in detail before^26^). The OASIS-D study was conducted between October 2020 and December 2024 at eight University psychiatric inpatient sites: Charité-St. Hedwig Hospital; Charité-Campus Mitte, Charité-Campus Benjamin Franklin, Ludwig-Maximilians-University Munich, University Hospital Frankfurt, University of Cologne, University Hospital Carl Gustav Carus Dresden, and Central Institute of Mental Health Mannheim. The study was approved (EA2/061/20) by the Ethics Committee of the Charité-Universitätsmedizin Berlin, which was the coordinating center, and by the Ethics Committees of the eight recruitment sites. For the current Part-2 of the OASIS-D study, written informed consent was obtained.

### Participants

#### Patient inclusion criteria

Inclusion criteria comprised: (i) males, females, or diverse; (ii) aged 18–75 years old; (iii) admission to a psychiatric inpatient facility; (iv) clinical diagnosis according to ICD-10 of a single or recurrent moderate (F32.1; F33.1) or severe depressive episode without (F32.2; F33.2) or with psychotic features (F32.3; F33.3); (v) presence of clinically assessed suicidality persisting for ≥48 hours following admission; and (vi) sufficient German language proficiency to comprehend the study’s aims and procedures.

#### Patient exclusion criteria

Exclusion criteria comprised: (i) psychiatric inpatients <18 or >75 years old; (ii) depressive symptoms secondary to a medical condition or substance use; (iii) presence of serious physical health issues that would interfere with study participation; (iv) insufficient comprehension of the study procedures; (v) inability to provide informed consent; and (vi) legal incapacity or limited legal competence preventing valid consent.

#### Treatment and assessments

Treatment was naturalistic as per clinicians’ and patients’ choice. The data collected included sociodemographic, illness, and treatment data (ie, age, sex, body mass index [BMI], family psychiatric disorders, education/work, pathways to admission, psychiatric ICD-10 F-diagnoses, illness duration, number of depressive episodes, current and lifetime suicidal thoughts and behaviors, psychiatric emergency admission, current and previous psychotropic treatments, antidepressant treatment resistance and nonadherence, substance use). Psychiatric diagnoses were based in clinical assessment as well as via research interview with the Mini-International Neuropsychiatric Interview (MINI).^27^ The clinical assessments were part of usual care by the treating clinician, who in German hospitals is typically a resident physician or trained psychiatrist, each under the supervision of a senior attending. Suicidality was assessed with the Sheehan-Suicidality Tracking Scale (S-STS).^28^ The data were collected at ≥48 h after inpatient admission (T1) and were transmitted from the study sites to the Charité coordinating center in pseudonymized form at an individual level via electronic case report form.

#### Clinical versus MINI-based diagnoses of MDD

Clinical diagnoses of MDD and psychiatric comorbidities were made based on interviews conducted by clinicians responsible for the admission of the patient according to local standards, applying ICD-10 criteria. The structured research diagnoses were based on the MINI and conducted by experienced psychiatrist or psychologists, designated by the site principal investigator, who underwent a standardized training via a MINI training video. The MINI is aligned with Diagnostic and Statistical Manual of Mental Disorders, Fifth Edition (DSM-5)^29^ criteria, but one adaptation was made to ensure full compatibility with ICD-10 standards used in clinical practice: the MINI item assessing fatigue or loss of energy (question A5) was considered as a potential third core MDD symptom, in addition to depressed mood and loss of interest, to meet the ICD-10 requirement of ≥2 out of three core criteria for MDD. This adaptation ensured that any “misdiagnosis” was not simply due to diagnostic discrepancy between ICD-10 and DSM-5.

#### Rater training

To minimize potential sources of bias, standardized diagnostic instruments (eg, MINI, S-STS) and criteria (ICD-10) were used to enhance diagnostic accuracy and reduce inter-rater variability. Moreover, clinical interviews were conducted by trained raters using standardized procedures, and all raters underwent a formal rater training either in person or watching recorded training videos.

#### Data analysis

The following analyses were conducted: First, patients were divided into two subgroups based on whether or not the clinical diagnosis of MDD was confirmed by the structured research-based MINI. Second, univariate comparisons were performed to identify differences in baseline variables between these two groups (univariate chi-square tests [effect size: Փ] and logistic regression analyses). Third, backward multivariable logistic regression analyses were used to identify independent predictors of false-positive MDD diagnoses, excluding research-based primary diagnoses trumping false-positive MDD that were observed exclusively in discordant cases, lacking no variability for model estimation. Univariate results were not controlled for multiple comparisons, as these were exploratory to yield potentially relevant candidate variables that were significantly associated with the outcome of interest. These candidate variables were then entered into multivariable regression models, retaining in the final model only factors that were independently significantly related to the outcome, not requiring controlling for multiple testing in that analysis. Fourth, we subdivided psychiatric comorbidities into MINI-based concordant and discordant clinical diagnoses. Fifth, Cohen’s kappa statistics were calculated to assess agreement between clinically diagnosed psychiatric comorbidities with MINI-based diagnoses (kappa score interpretation^30^: <0 = poor, 0–0.20 = slight, 0.21–0.40 = fair, 0.41–0.60 = moderate, 0.61–0.80 = substantial, >0.80 = almost perfect). Sixth, backward multivariable logistic regression analyses were used to identify independent demographic, illness, and treatment correlates of specific discordant psychiatric comorbidities. Due to collinearity of variables related to number and type of psychiatric comorbidities, two different models were used, including either (i) the total number of comorbidities or (ii) individual comorbid diagnoses (each based on MINI or clinical diagnosis).

Only variables with available data for ≥67% of the total sample were included in the initial variable selection for multivariable models. When variables capturing the same domain were collinear, the variable with greater statistical association with the dependent variable in the univariate analysis was chosen. All analyses were conducted two-sided with alpha = 0.05, using R version 4.4.3 (R Core Team, 2024).^31^

## Results

### Study population

Altogether, 401 inpatients (median age = 33.0 years [IQR = 26.0–47.0]; females = 57.1%, Hollingshead^32^ score = 37.0 [IQR = 21.0, 48.0], median = middle class) were analyzed. Regarding patient admission pathways, most patients were self-admitted (52.5%), followed by referrals from outpatient mental health practitioners (17.5%), family/friends (12.0%), police/ambulance (8.8%), and inpatient care providers (6.0%). Over half (52.4%) of the patients were admitted due to a psychiatric emergency, the vast majority (95.2%) being related to suicidality (Table 1). For additional categorical patient and admission characteristics, see Table 1.

**Table 1.** Categorical baseline patient and admission characteristics of psychiatrically hospitalized adults with major depressive disorder Table 1. long description.

Characteristic	Total (*n* = 401)	Concordant clinical-to-research MDD diagnosis (*n* = 359)	Discordant clinical-to-research MDD diagnosis (*n* = 42)	*p*-value	Effect size,Փ(95% CI)
Demographic characteristics					
Sex					
Female, *n* (%)	229 (57.1)	210 (58.5)	19 (45.2)	0.236	0.08 (0.00, 0.18)
Male	171 (42.6)	148 (41.2)	23 (54.8)		
Diverse	1 (0.3)	1 (0.3)	0 (0.0)		
Family history of psychiatric disorders	288 (76.2)	260 (76.2)	28 (75.7)	0.938	0.00 (0.00, 0.10)
Socioeconomic status					
Currently employed	179 (45.9)	162 (46.6)	17 (40.5)	0.455	0.04 (0.00, 0.14)
Highest educational attainment					
Completed studies/vocational training	182 (45.4)	165 (46.0)	17 (40.5)	0.408	0.12 (0.02, 0.22)
High school diploma/technical college entrance qualification	97 (24.2)	86 (24.0)	11 (26.2)		
Intermediate school degree (at least 10 years) or equivalent	40 (10.0)	38 (10.6)	2 (4.76)		
Incomplete studies/vocational training	34 (8.5)	27 (7.5)	7 (16.7)		
Lower secondary education (at least 9 years)	34 (8.5)	31 (8.6)	3 (7.1)		
Doctorate/further postgraduate qualifications (eg, medical specialist)/dual degrees	12 (3.0)	10 (2.8)	2 (4.8)		
No degree	2 (0.5)	2 (0.6)	0 (0.0)		
Occupation [n = 391]					
Housekeeper, student, agricultural worker, unskilled laborer	100 (25.6)	89 (25.5)	11 (26.2)	0.088	0.19 (0.09, 0.29)
Clerical and sales employees, small agricultural landowners	82 (21.0)	75 (21.5)	7 (16.7)		
Small business owners, farmers, managers, mid-grade civil servant	55 (14.1)	50 (14.3)	5 (11.9)		
Technical employee, small business owner (eg, independent tradesman)	41 (10.5)	38 (10.9)	3 (7.1)		
Unskilled worker	32 (8.2)	29 (8.3)	3 (7.1)		
Medium business owners, higher-grade civil servant	30 (7.7)	26 (7.5)	4 (9.5)		
Craftsman, tenant farmers, lower-grade civil servant	23 (5.9)	20 (5.7)	3 (7.1)		
Machine operator and semi-skilled worker	16 (4.1)	15 (4.3)	1 (2.4)		
Executive manager, large business owner, high-grade civil servant	12 (3.1)	7 (2.0)	5 (11.9)		
Inpatient admission context					
Referral context [*n* = 400]					
Self-referral	210 (52.5)	192 (53.5)	18 (43.9)	0.001	0.23 (0.13, 0.33)
Outpatient therapist	70 (17.5)	64 (17.8)	6 (14.6)		
Family/friends	48 (12.0)	44 (12.3)	4 (9.8)		
Police/ambulance	35 (8.8)	32 (8.91)	3 (7.3)		
Other clinic	24 (6.0)	20 (5.6)	4 (9.8)		
Other referral context	13 (3.3)	7 (1.95)	6 (14.6)		
Psychiatric emergency at time of admission	209 (52.4)	189 (52.9)	20 (47.6)	0.514	0.03 (0.00, 0.13)
Reason for psychiatric emergency [*n* = 209]					
Suicidality	199 (49.6)	180 (50.1)	19 (45.2)	0.856	0.06 (0.00, 0.20)
Acute agitation	6 (1.5)	5 (1.4)	1 (2.4)		
Psychosis	2 (0.5)	2 (0.6)	0 (0.0)		
Other self-harm (eg, stupor, refusal of eat)	2 (0.5)	2 (0.6)	0 (0.0)		
Psychiatric emergency due to suicidality in the last 24 h	28 (7.5)	28 (8.2)	0 (0.0)	0.078	0.09 (0.00, 0.19)

Altogether, 57.9% of the patients had ≥1 prior lifetime hospitalization for a MDD (Table 2). Regarding outpatient care, 64.5% of the patients had received outpatient psychiatric/psychotherapeutic care within the last 6 months prior to admission, and 81.6% had been in outpatient psychiatric/psychotherapeutic care at some point of their life. Altogether, 87.3% had a severe depressive episode, 12.7% a moderately severe depressive episode, while severe depressive episodes with psychotic symptoms were rare (3.0%). Almost three quarters (73.8%) had a recurrent depressive episode and 22.9% had treatment-resistant depression. A little less than half (46.6%) of the patients had clinically diagnosed psychiatric comorbidities, consisting mostly of alcohol or other substance use disorders (ASUDs) (11.0%), posttraumatic stress disorder (10.7%), personality disorders (9.0%), and anxiety disorders (8.2%). Nearly two-thirds (68.3%) of the patients were prescribed psychotropic medications at the time of admission. The most commonly prescribed psychotropic medications were selective serotonin reuptake inhibitors (SSRI) (24.4%), followed by second-generation antipsychotics (23.9%), serotonin-norepinephrine reuptake inhibitors (SNRI) (19.2%), noradrenergic and specific serotonergic antidepressant (16.2%), and anxiolytics or hypnotics (16.0%). Furthermore, 5.5% reported electroconvulsive therapy during their lifetime (Table 2).

**Table 2.** Categorical baseline patient illness and treatment characteristics of psychiatrically hospitalized adults with major depressive disorder Table 2. long description.

Characteristic	Total (*n* = 401)	Concordant clinical-to-research MDD diagnosis (*n* = 359)	Discordant clinical-to-research MDD diagnosis (*n* = 42)	*p*-value	Effect size,Փ(95% CI)
Clinical MDD diagnoses					
Diagnosis of MDD					
Severe without psychotic symptoms	338 (84.3)	303 (84.4)	35 (83.3)	0.926	0.02 (0.00, 0.12)
Moderate	51 (12.7)	45 (12.5)	6 (14.3)		
Severe with psychotic symptoms	12 (3.0)	11 (3.1)	1 (2.4)		
First MDD (vs. recurrent MDD)	105 (26.2)	93 (25.9)	12 (28.6)	0.710	0.00 (0.00, 0.10)
Treatment-resistant depression	87 (22.9)	81 (23.8)	6 (15.0)	0.209	0.06 (0.00, 0.16)
Any clinically diagnosed comorbid psychiatric disorder	173 (46.6)	159 (47.5)	14 (38.9)	0.102	0.12 (0.02, 0.22)
Alcohol and substance use disorder	44 (11.0)	39 (10.9)	5 (11.9)	0.838	0.01 (0.00, 0.11)
Personality disorder	36 (9.0)	34 (9.5)	2 (4.8)	0.312	0.05 (0.00, 0.15)
Phobic anxiety disorder and other anxiety disorders	33 (8.2)	32 (8.9)	1 (2.4)	0.145	0.07 (0.00, 0.17)
Attention-deficit/hyperactivity disorder	13 (3.2)	12 (3.3)	1 (2.4)	0.739	0.02 (0.00, 0.12)
Other comorbid disorder	95 (55.9)	88 (56.4)	7 (50.0)	0.644	0.04 (0.00, 0.19)
Any comorbid psychiatric disorder as identified by the research-based MINI interview					
Bipolar disorder	19 (4.7)	0 (0.0)	19 (45.2)	<0.001	0.65 (0.58, 0.72)
Substance use-related disorders	114 (28.4)	93 (25.9)	21 (50.0)	0.001	0.16 (0.06, 0.26)
Anxiety disorders	181 (45.1)	167 (46.5)	14 (33.3)	0.104	0.08 (0.00, 0.18)
Disorder as a result of physical illness or substance use	10 (2.5)	8 (2.3)	2 (4.9)	0.312	0.05 (0.00, 0.15)
Subsyndromal depression	9 (2.2)	0 (0.0)	9 (21.4)	<0.001	0.44 (0.35, 0.53)
Suicidality					
Suicidality, current [*n* = 398]					
Passive	159 (39.9)	138 (38.8)	21 (50.0)	0.119	0.14 (0.04, 0.24)
Active without plan	83 (20.9)	80 (22.5)	3 (7.14)		
Active with plan	82 (20.6)	75 (21.1)	7 (16.7)		
Active with plan and intention	51 (12.8)	43 (12.1)	8 (19.0)		
No suicidal thoughts	23 (5.8)	20 (5.6)	3 (7.14)		
Suicide attempt, current (<2 weeks before admission)	71 (17.8)	60 (16.8)	11 (26.2)	0.133	0.08 (0.00, 0.18)
Suicidality, lifetime					
Active with plan and intention, lifetime	233 (58.8)	209 (58.9)	24 (58.5)	0.598	0.07 (0.00, 0.17)
Passive or active suicidality without suicide attempt	155 (39.1)	138 (38.9)	17 (41.5)		
No suicidal thoughts/attempts	8 (2.0)	8 (2.3)	0 (0.0)		
Suicide attempt, lifetime	191 (47.9)	168 (47.1)	23 (54.8)	0.345	0.05 (0.00, 0.15)
Psychotropic medication (persons), *n* (%)					
Any psychotropic medication	274 (68.3)	247 (68.8)	27 (64.3)	0.552	0.03 (0.00, 0.13)
Antidepressants	241 (60.1)	215 (59.9)	26 (61.9)	0.801	0.01 (0.00, 0.11)
SSRIs	98 (24.4)	86 (24.0)	12 (28.6)	0.510	0.03 (0.00, 0.13)
SNRIs	77 (19.2)	70 (19.5)	7 (16.7)	0.659	0.02 (0.00, 0.12)
NaSSA	65 (16.2)	60 (16.7)	5 (11.9)	0.424	0.04 (0.00, 0.14)
Tricyclic antidepressants	10 (2.5)	8 (2.2)	2 (4.8)	0.319	0.05 (0.00, 0.15)
MAOI-Inhibitors	4 (1.0)	4 (1.1)	0 (0.0)	0.492	0.03 (0.00, 0.13)
Others					
Bupropion	32 (8.0)	31 (8.6)	1 (2.4)	0.157	0.07 (0.00, 0.17)
Trazodone	24 (6.0)	23 (6.4)	1 (2.4)	0.298	0.05 (0.00, 0.15)
Agomelatine	13 (3.2)	12 (3.3)	1 (2.4)	0.739	0.02 (0.00, 0.12)
Ketamine	1 (0.3)	1 (0.3)	0 (0.0)	0.732	0.02 (0.00, 0.12)
Opipramol	1 (0.3)	1 (0.3)	0 (0.0)	0.732	0.02 (0.00, 0.12)
Mood stabilizer	34 (8.5)	31 (8.6)	3 (7.1)	0.743	0.02 (0.00, 0.12)
Lithium	29 (7.3)	26 (7.2)	3 (7.1)	0.981	0 (0.00, 0.1)
Antiepileptics	12 (3.0)	12 (3.3)	0 (0.0)	0.229	0.06 (0.00, 0.16)
Antipsychotics	105 (26.2)	98 (27.3)	7 (16.7)	0.138	0.07 (0.00, 0.17)
First-generation antipsychotics	8 (2.0)	7 (2.0)	1 (2.4)	0.850	0.01 (0.00, 0.11)
Second-generation antipsychotics	96 (23.9)	90 (25.1)	6 (14.3)	0.121	0.08 (0.00, 0.18)
Anxiolytics/hypnotics	64 (16.0)	58 (16.2)	6 (14.3)	0.754	0.02 (0.00, 0.12)
Other psychotropic medications	19 (4.7)	18 (5.0)	1 (2.4)	0.447	0.04 (0.00, 0.14)
Psychotropic medication nonadherence (6 months before)	62 (15.8)	57 (16.2)	5 (12.2)	0.502	0.03 (0.00, 0.13)
Nonadherence to psychotropic medication, lifetime	107 (27.6)	96 (27.7)	11 (26.8)	0.910	0.01 (0.00, 0.11)
Previous treatment					
Previous inpatient treatment for depressive episodes	228 (57.9)	207 (58.8)	21 (50.0)	0.275	0.06 (0.00, 0.16)
Outpatient psychotherapeutic/psychiatric treatment, last 6 months before admission	256 (64.5)	232 (65.4)	24 (57.1)	0.293	0.05 (0.00, 0.15)
Outpatient psychotherapeutic/psychiatric treatment, lifetime	324 (81.6)	288 (81.1)	36 (85.7)	0.468	0.04 (0.00, 0.14)
Electroconvulsive therapy, lifetime	22 (5.5)	22 (6.2)	0 (0.0)	0.098	0.08 (0.00, 0.18)

At admission, 94.2% of patients reported at least some suicidality, including passive suicidal thoughts (39.9%), active suicidal thoughts without a plan (20.9%), active suicidal thoughts with a plan (20.6%), and active suicidal thoughts with both plan and intent (12.8%). Over their lifetime, nearly all patients (98.0%) had experienced suicidal thoughts, comprising passive or active thoughts without an attempt (39.1%) and active suicidal thoughts with a plan and intent (58.8%). Overall, nearly one-fifth (17.8%) of the patients had attempted suicide within two weeks prior to admission, and almost half (47.9%) reported a lifetime history of suicide attempts (Table 2).

Information about treatments received at admission is displayed in Table 3.

**Table 3.** Continuous baseline patient demographic, treatment, and illness characteristics of psychiatrically hospitalized adults with major depressive disorder Table 3. long description.

Characteristic	Total (*n* = 401)	Concordant clinical-to-research MDD diagnosis (*n* = 359)	Discordant clinical-to-research MDD diagnosis (*n* = 42)	*p*-value	Beta coefficient (β)	Odds ratio (95% CI)
Age, median (IQR)	33.0 (26.0, 47.0)	33.0 (25.0, 48.0)	34.5 (27.0, 43.0)	0.887	0.00	1.00 (0.98, 1.02)
BMI (kg/m^2^), median (IQR)	24.2 (21.4, 28.7)	24.3 (21.4, 29.1)	23.50 (21.4, 25.9)	0.118	0.05	1.05 (0.99, 1.12)
Hollingshead socioeconomic status	37.0 (21.0, 48.0)	37.0 (20.0, 48.0)	41.00 (22.0, 53.0)	0.343	−0.01	0.99 (0.97, 1.01)
Number of depressive episodes, mean (SD)	3.4 (2.8)	3.3 (2.8)	3.7 (2.4)	0.408	−0.04	0.96 (0.87, 1.07)
Number of comorbid diagnoses, mean (SD)	0.7 (1.0)	0.7 (1.1)	0.5 (0.9)	0.262	0.21	1.23 (0.88, 1.85)
Number of suicide attempts, mean (SD)	2.1 (1.8)	2.1 (1.8)	1.9 (1.3)	0.560	0.09	1.09 (0.85, 1.55)
Number of psychotropic medications, mean (SD)	1.4 (1.4)	1.5 (1.4)	1.2 (1.2)	0.158	0.19	1.21 (0.94, 1.59)
Number of psychotropic medication in person with at least one drug	2.1 (1.1)	2.1 (1.1)	1.8 (1.0)	0.146	0.30	1.36 (0.92, 2.11)
Number of antidepressants, mean (SD)	0.8 (0.8)	0.8 (0.8)	0.7 (0.6)	0.280	0.24	1.27 (0.84, 1.97)
Number of antidepressants in person with at least one drug	1.2 (0.7)	1.2 (0.7)	1.1 (0.4)	0.337	0.30	1.35 (0.74, 2.51)
Number of mood stabilizers, mean (SD)	0.1 (0.3)	0.1 (0.3)	0.1 (0.3)	0.710	0.23	1.26 (0.44, 5.29)
Number of mood stabilizers in person with at least one drug	0.1 (0.4)	0.1 (0.4)	0.1 (0.3)	0.792	0.16	1.18 (0.40, 5.04)
Number of antipsychotics, mean (SD)	0.3 (0.5)	0.3 (0.6)	0.2 (0.4)	0.100	0.65	1.92 (0.95, 4.61)
Number of antipsychotics in person with at least one drug	0.4 (0.6)	0.5 (0.6)	0.3 (0.5)	0.113	0.66	1.94 (0.91, 4.82)

### Disagreement (true positive vs. false positive) regarding clinical MDD diagnosis

A clinical diagnosis of MDD was confirmed in 89.5% patients with the MINI (true positive). However, 10.5% were false positive, in that the MINI yielded a different primary psychiatric diagnosis, consisting predominantly of BD (45.2%), including bipolar-I disorder (28.6%) and bipolar-II disorder (16.7%). Additional missed primary diagnoses included ASUDs (23.8), subsyndromal depression (16.7%) (ie, not meeting full ICD-10 criteria for MDD), anxiety disorders (9.5%) (2 each with panic disorder and generalized anxiety disorder), and secondary depression attributed to physical illness/substance use (4.8%) (Table 4).

**Table 4.** Diagnostic Agreement Between Clinical Routine and Research-Based Diagnoses: Cross-Tabulation of Primary and Comorbid Diagnoses in Psychiatrically Hospitalized Adults with Major Depressive Disorder Table 4. long description.

Primary research-based diagnosis, *n* (%)	MINI-based diagnoses (*n* = 401)	Clinical diagnoses (*n* = 401)	True positive (*n* = 359)	False negative (*n* = 42)
Major depressive disorder	359 (89.5)	401 (100)	359 (100)	0 (0.0)
Bipolar disorder	19 (4.7)	0 (0.0)	0 (0.0)	19 (45.2)
Bipolar I disorder	12 (3.0)	0 (0.0)	0 (0.0)	12 (28.6)
Bipolar II disorder	7 (1.7)	0 (0.0)	0 (0.0)	7 (16.7)
Substance use disorder	10 (2.5)	0 (0.0)	0 (0.0)	10 (23.8)
Subsyndromal major depressive disorder	7 (1.7)	0 (0.0)	0 (0.0)	7 (16.7)
Anxiety disorder	4 (1.0)	0 (0.0)	0 (0.0)	4 (9.5)
Secondary depression as a result of physical illness or substance use	2 (0.5)	0 (0.0)	0 (0.0)	2 (4.8)

Research-based comorbid diagnoses, *n* (%)	MINI-based diagnoses (*n* = 401)	Clinical diagnoses (*n* = 401)	True positive	False positive	True negative	False negative	Cohen’s Kappa (95% CI)	Standard error	*p*-value
			(*n* = 401)						
Posttraumatic stress disorder	95 (23.7)	43 (10.7)	34 (8.5)	9 (2.2)	297 (74.1)	61 (15.2)	0.40 (0.30, 0.51)	0.06	<0.001
Eating disorder	29 (7.2)	14 (3.5)	9 (2.2)	5 (1.2)	367 (91.5)	20 (5.0)	0.39 (0.16, 0.62)	0.12	0.001
Substance use disorder	114 (28.4)	44 (11.0)	32 (8.0)	12 (3.0)	275 (68.6)	82 (20.4)	0.29 (0.17, 0.42)	0.06	<0.001
Obsessive-compulsive disorder	35 (8.7)	12 (3.0)	7 (1.7)	5 (1.2)	361 (90.0)	28 (7.0)	0.27 (0.02, 0.51)	0.12	0.031
Anxiety disorder	181 (45.1)	30 (7.5)	29 (7.2)	1 (0.2)	219 (54.6)	152 (37.9)	0.17 (0.06, 0.27)	0.05	0.001
Antisocial personality disorder	21 (5.2)	0 (0.0)	0 (0.0)	0 (0.0)	380 (94.8)	21 (5.2)	0.0	-	-

### Univariate correlates of a true-positive versus false-positive clinical MDD diagnosis

In univariate analyses comparing the true-positive versus false-positive clinical MDD diagnostic groups, only four baseline variables differed statistically significantly between the two (ie, referral context; *p* = 0.001; *Փ* =0.23) and three research-based diagnoses (ie, BD; *p* < 0.001; *Փ* =0.65), subsyndromal depression (*p* < 0.001; *Փ* =0.44), and ASUD (*p* = 0.001; *Փ* 0.16) groups (Tables 1–3).

### Multivariable backward regression correlates of true-positive versus false-positive clinical MDD diagnosis

The multivariable regression analyses, which excluded primary, false-negative research-based MINI diagnoses, which were exclusively present in the false-positive MDD cases, yielded three independent statistically significantly variables related to false-positive MDD diagnoses: ASUDs (OR = 3.16, p = 0.001), current antidepressant prescription (OR = 2.97, p = 0.027), and fewer psychotropic medications (OR = 1.67, p = 0.017) (*R* ^2^=0.08) (Table 5).

**Table 5.** Final Multivariable Regression Models of Independent Correlates that are Significantly Associated with Discordant Diagnoses of a Research-Based Versus Clinical Diagnosis of Major Depressive Disorder or Comorbidities in Psychiatrically Hospitalized Adults Table 5. long description.

False-positive clinical diagnosis of MDD		Predictor	OR (95% CI)	Standard error	*p*-value	Nagelkerke’s *R* ^2^
Non-MDD research-based diagnoses (excluding as predictors 100% false-negative disorders)		Substance use disorder (MINI)	3.16 (1.62, 6.17)	0.34	0.001	0.08
		Antidepressant medication	2.97 (1.15, 8.01)	0.49	0.027	
		Lower number of psychotropic medications	1.67 (1.13, 2.63)	0.22	0.017	

Discordant psychiatric comorbidity in patients with a clinical diagnosis of MDD	Level of analysis (number of comorbidities or specific individual comorbidities)	Predictor	OR (95% CI)	Standard error	*P*-value	Nagelkerke’s *R* ^2^
Anxiety disorders	Number of comorbidities	Higher number of comorbidities (MINI)	3.46 (2.72, 4.53)	0.13	<0.001	0.46
		Greater psychotropic medication adherence in the last 6 months	2.89 (1.39, 6.36)	0.39	0.006	
		Treatment-resistant depression	2.53 (1.39, 4.62)	0.30	0.002	
		Lower number of clinical diagnoses-based comorbidities (clinical diagnoses)	2.41 (1.80, 3.35)	0.16	<0.001	
	Individual comorbidities	Obsessive-compulsive disorder (MINI interview)	2.05 (1.02, 4.18)	0.36	0.043	0.01
Antisocial personality disorder	Number of comorbidities	Absent of antidepressant prescriptions	10.80 (2.03, 70.11)	0.89	0.008	0.44
		Absent of psychiatric emergency at admission	5.37 (1.68, 20.09)	0.62	0.007	
		Sex (male)	3.86 (1.31, 12.55)	0.57	0.018	
		Higher number of comorbidities (MINI)	3.22 (2.20, 5.21)	0.21	<0.001	
		Higher number of psychotropic medications	1.99 (1.12, 3.63)	0.30	0.020	
	Individual comorbidities	Eating disorders (MINI)	4.90 (1.32, 16.44)	0.63	0.012	0.18
		Sex (male)	4.53 (1.67, 14.30)	0.54	0.005	
		Anxiety disorders (MINI)	3.00 (1.15, 8.85)	0.51	0.032	
		Nonadherence in the last 6 months	2.72 (1.00, 7.17)	0.50	0.044	
Posttraumatic stress disorder	Number of comorbidities	Sex (female)	2.12 (1.21, 3.83)	0.29	0.010	0.12
		Number of comorbidities (MINI)	1.66 (1.26, 2.20)	0.14	<0.001	
		Higher BMI at admission	1.05 (1.01, 1.09)	0.02	0.029	
		Lower socioeconomic status	1.02 (1.01, 1.04)	0.01	0.030	
	Individual comorbidities	Substance use disorder (MINI)	2.35 (1.35, 4.10)	0.28	0.002	0.07
		Sex (female)	1.96 (1.13, 3.51)	0.29	0.020	
		Antipsychotic medications	1.94 (1.10, 3.38)	0.29	0.021	
Eating disorders	Number of comorbidities	Sex (female)	6.55 (2.16, 25.53)	0.62	0.002	0.30
		Number of comorbidities (MINI)	2.08 (1.57, 2.80)	0.15	<0.001	
		Number of comorbidities (clinical diagnoses)	1.54 (1.09, 2.16)	0.17	0.011	
	Individual comorbidities	Sex (female)	3.25 (1.28, 9.97)	0.51	0.021	0.08
		Anxiety disorders (MINI)	2.80 (1.21, 7.03)	0.44	0.021	
Obsessive-compulsive disorder	Number of comorbidities	Psychotropic adherence in the last 6 months	4.95 (1.43, 24.29)	0.71	0.024	0.23
		Treatment-resistant depression	2.46 (1.03, 5.75)	0.44	0.039	
		Number of comorbidities (MINI)	2.10 (1.63, 2.75)	0.13	<0.001	
		Lower number of psychotropic medications	1.39 (1.02, 1.95)	0.17	0.048	
	Individual comorbidities	Anxiety disorders (MINI)	4.10 (1.86, 9.97)	0.42	0.001	0.10
		Number of depressive episodes	1.12 (1.00, 1.23)	0.05	0.034	
Alcohol and substance use disorders	Number of comorbidities	Lower number of previous depressive episodes	1.97 (1.14, 3.39)	0.28	0.015	0.21
		Number of comorbidities (MINI)	1.90 (1.58, 2.27)	0.09	<0.001	
		Lower number of comorbidities (clinical diagnoses)	1.54 (1.17, 2.08)	0.14	0.003	
	Individual comorbidities	Nonadherence in the last 6 months	1.88 (1.04, 3.32)	0.29	0.032	0.02

### Discordance between clinical and MINI-based psychiatric comorbidities

Cross-tabulation analyses using Cohen’s *κ* showed variable levels of agreement between MINI-based and clinical routine diagnoses for psychiatric comorbidities in patients with a clinical MDD diagnosis. The diagnostic discordance was highest for antisocial personality disorder, which was not diagnosed at all in routine care but was present as per MINI in 21 patients (5.2%) ( *κ* =0.00). The next highest diagnostic discordance was found for anxiety disorders (false-negative: *n* = 152 [37.9%], false-positive: *n* = 1 [0.2%]; *κ* =0.17, *p* = 0.001), followed by OCD (false-negative: *n* = 28 [7.0%], false-positive: *n* = 5 [1.2%]; *κ* =0.27, *p* = 0.031), ASUDs (false-negative: *n* = 82 [20.4%], false-positive: *n* = 12 [3.0%]; *κ* =0.29, *p* < 0.001), eating disorders (false-negative: *n* = 20 [5.0%], false-positive: *n* = 5 [1.2%]; *κ* =0.39, *P* = 0.001), and posttraumatic stress disorder (false-negative: *n* = 61 15.2%, false-positive: *n* = 9 2.2%; *κ* =0.40, *P* < 0.001) (Table 4).

### Multivariable backward regression correlates of concordant versus discordant clinical-to-research ICD-10 comorbid diagnoses

Due to high collinearity and different levels of information for the total number of comorbidities and individual comorbid diagnoses, two separate backward multivariable regression analysis were conducted using either one or the other predictor variables.

Discordant clinical anxiety disorder diagnoses were significantly associated in model 1 with higher number of MINI-based comorbidities (OR = 3.46, *P* < 0.001), greater psychotropic medication adherence in the last 6 months (OR = 2.89, *P* = 0.006), treatment-resistant depression (OR = 2.53, *P* = 0.002), and lower number of clinical diagnoses-based comorbidities (OR = 2.41, *P* < 0.001) (*R* ^2^= 0.46). In model 2, solely OCD (OR = 2.05, *P* = 0.043) remained as a significant predictor of diagnostic discordance (*R* ^2^= 0.01) (Table 5).

Discordant clinical antisocial personality disorder diagnoses were significantly associated in model 1 with absent antidepressant prescriptions (OR = 10.80, *P* = 0.008), absent psychiatric emergency at admission (OR = 5.37, *P* = 0.007), male sex (OR = 3.86, *P* = 0.018), higher number of MINI-based comorbidities (OR = 3.22, *P* < 0.001), and higher number of psychotropic medications (OR = 1.99, *P* = 0.020) (*R* ^2^= 0.44). In model 2, discordant antisocial personality disorder diagnoses were significantly predicted by MINI-based eating disorders (OR = 4.90, *P* = 0.012), male sex (OR = 4.53, *P* = 0.005), MINI-based anxiety disorders (OR = 3.00, *P* = 0.032), and greater psychotropic medication nonadherence in the last 6 months (OR = 2.72, *P* = 0.044) (*R* ^2^= 0.18) (Table 5).

Discordant clinical PTSD diagnosis were significantly associated in model 1 with female sex (OR = 2.12, *P* = 0.010), higher number of MINI-based comorbidities (OR = 1.66, *P* < 0.001), higher admission BMI (OR = 1.05, *P* = 0.029), and lower socioeconomic status (OR = 1.02, *P* = 0.030) (*R* ^2^ = 0.12). In model 2, discordant PTSD diagnoses were significantly predicted by MINI-based ASUDs (OR = 2.35, *P* = 0.002), female sex (OR = 1.96, *P* = 0.020), and antipsychotic prescriptions (OR = 1.94, *P* = 0.021) (*R^2^* = 0.07) (Table 5).

Discordant clinical eating disorder diagnoses were significantly associated in model 1 with female sex (OR = 6.55, *P* = 0.002), higher number of MINI-based comorbidities (OR = 2.08, *P* < 0.001), and higher number of clinical diagnosis-based comorbidities (OR = 1.54, *P* = 0.011) (*R* ^2^ = 0.30). In model 2, discordant eating disorder diagnoses were significantly predicted by female sex (OR = 3.25, *P* = 0.021) and MINI-based anxiety disorders (OR = 2.80, *P* = 0.021) (*R* ^2^ = 0.08) (Table 5).

Discordant clinical OCD diagnoses were significantly associated in model 1 with psychotropic medication adherence in the last 6 months (OR = 4.95, *P* = 0.024), treatment-resistant depression (OR = 2.46, *P* = 0.039), a higher number of MINI-based psychiatric comorbidities (OR = 2.10, *P* < 0.001), and a lower number of psychotropic medications (OR = 1.39, *P* = 0.048) (*R* ^2^= 0.23). In model 2, discordant OCD diagnoses were significantly predicted by MINI-based anxiety disorders (OR = 4.10, *P* = 0.001) and higher lifetime number of depressive episodes (OR = 1.12, *P* = 0.034) (*R* ^2^= 0.10) (Table 5).

Discordant clinical ASUDs diagnoses were significantly associated in model 1 psychotropic medication with a lower lifetime number of depressive episodes (OR = 1.97, *P* = 0.015), higher MINI-based numbers of comorbidities (OR = 1.90, *P* < 0.001), and a lower number of clinical diagnosis-based comorbidities (OR = 1.54, *P* = 0.003) (*R* ^2^= 0.21). In model 2, solely psychotropic medication nonadherence in the past 6 months (OR = 1.88, *P* = 0.032) remained as a significant predictor of diagnostic discordant ASUDs (*R* ^2^ = 0.02) (Table 5).

## Discussion

The present study showed that: (i) in ~10% of patients, the clinical MDD diagnosis was unconfirmed via research-interview; (ii) half of these false-positive cases had BD per MINI; one quarter had a primary ASUDs, one out of six had subsyndromal depressive syndromes, and one out of ten had primary anxiety disorders; (iii) in a multivariable analysis excluding missed primary psychiatric diagnoses only present among false-positive MDD cases, three independent correlates of inaccurate MDD diagnoses were identified, including research MINI-diagnosed ASUDs, more prescribed antidepressants, and fewer prescribed psychotropic medications (*r* ^2^= 8%); (iv) diagnostic concordance between clinical and MINI-based comorbidities was limited across all comorbid psychiatric diagnoses, with fair agreement for posttraumatic stress disorder ( *κ* =0.40), eating disorders ( *κ* =0.39), ASUDs ( *κ* =0.29), and OCD ( *κ* =0.27), slight agreement for anxiety disorders ( *κ* =0.17), and complete lack of clinical diagnoses for antisocial personality disorder ( *κ* =0.00); and (v) in additional multivariable analyses focusing on discordant comorbidities, predictors of discordance were largely comorbidity-related and varied by comorbid diagnoses, but predominantly, more MINI-based comorbidities predicted diagnostic discordance, while more clinically diagnosed comorbidities predicted diagnostic concordance.

The 10% false-positive rate of clinically diagnosed versus research-interview-based MDD observed in our study of psychiatric inpatients is markedly lower than the 58% false-positive rate reported in a meta-analysis of 19 studies. However, the diagnoses in those studies were made by general practitioners in outpatient settings using unstructured clinical judgment, and the benchmark was not a research interview, but rather an estimated true MDD prevalence rate 20%.^15^ Similarly, higher false-positive rates of MDD diagnoses have been reported in psychiatric services, ranging from 24.6% to 55.2%.^16^ ^–^^20^ The higher reported false-positive rates may be explained by methodological and treatment setting differences, including clinical specialization of the diagnosing professionals (eg, university hospitals, outpatient units, or general practitioners),^15^ ^–^^18^ differences in inclusion criteria, type of research diagnostic assessment (eg, MINI, Structured Clinical Interview for DSM-5 Disorders-SCID-5, Schedules for Clinical Assessment in Neuropsychiatry (SCAN),^15^ ^,^^18^ ^–^^20^ and potential cross-national differences.^16^ ^–^^20^ In our study, several factors likely contributed to the comparatively low rate of false-positive MDD diagnoses, including the inpatient setting, university and teaching hospitals in urban regions, and the requirement of at least moderately severe depression with concurrent suicidality.

Among false-positive MDD cases in our sample, BD was the most frequently missed diagnosis, followed by ASUDs, subsyndromal depression, anxiety disorders, and depression secondary to physical illness/substance use. We identified only two studies that also reported all clinical diagnoses in false-positive cases of MDD. In one study conducted in an outpatient psychiatric setting, using the MINI as the gold standard, 37% of MDD diagnoses were misclassified. Among those, the most commonly missed diagnoses were anxiety disorders (48%), followed by psychotic disorders (7%), BD (5%), ASUDs (4%), and PTSD (3%).^16^ However, the study utilized the self-rated Penn State Psychiatry Clinical Assessment and Rating Evaluation System (PCARES), the results of which were available and useable to clinicians. In the other study, 24.6% of patients with a clinical diagnosis of first-episode MDD received a different diagnosis based on SCAN interview, including “other diagnoses” (8.8%: primarily anxiety, somatoform, or substance-related disorders), BD (3.3%), no psychiatric diagnosis (1.8%), schizophrenia/nonorganic psychosis (1.0%), and dysthymia (0.5%).^19^ Further studies, focusing only on the misdiagnosis of BD, have also reported a substantial proportion of false-positive MDD diagnoses, with misclassification rates ranging from 14% to 21%.^33^ ^–^^35^ Misdiagnosis of BD as MDD is a well-established clinical problem that is associated with treatment risks, particularly when antidepressants are prescribed without mood stabilizers, as well as increased treatment costs.^11^ ^,^^12^ ^,^^33^ ^,^^36^ ^–^^38^ Similarly, misdiagnosis of other disorders as MDD could also lead to less effective treatment outcomes.^39^ ^,^^40^ These findings suggest that the more comprehensive and structured diagnostic process offered by the MINI reveals misdiagnoses that stem from limited diagnostic depth in routine care.

Independent variables in the multivariable regression analysis that excluded diagnoses only present in false-positive MDD cases (ie, BD, subsyndromal depression, and depression due to physical illness and substance use), retained from the initial model only MINI-based ASUDs, current antidepressant medication prescriptions, and less prescribed psychotropic medications, showing weak explanatory power (*R* ^2^= 0.08). This limited model performance likely reflects the exclusion of core discordant conditions that explained of majority of misdiagnoses. ASUDs are commonly comorbid with MDD^41^ ^,^^42^ and can induce depressed mood^43^ and are often not disclosed, even when asked about directly, due to stigma.^44^ ^,^^45^ These factors may have contributed to misattributing ASUD as MDD. Similarly, an antidepressant prescription may have prompted a MDD diagnosis, although 10% among the false-positive MDD cases had a primary anxiety disorder, which may prompt antidepressant prescription. The higher number of psychotropic medications associated with false-positive MDD may well be due to more complex psychiatric primary and comorbid diagnoses, complicating accurate diagnostic procedures. Particularly, bipolar disorder has been associated to a mean number of 3–4 psychotropic medications due to treatment targets of depression, hypomania/mania, relapse prevention, and frequent comorbidities^41^ ^,^^42^ ^,^^46^ However, alternative explanations cannot be excluded, including the possibility that the association between antidepressant prescription and false-positive MDD diagnoses reflects reverse causation, whereby a prior prescription influenced the clinical diagnostic reasoning rather than resulting from it.

Furthermore, agreement between routine clinical and MINI-based comorbidities was low in our patients hospitalized for MDD. The fair level of concordance for ASUDs in our study ( *κ* =0.29) is consistent with or somewhat lower than results reported in two meta-analyses where the agreement ranged from fair-moderate ( *κ* =0.56; *κ* ≈0.30).^13^ ^,^^14^ For anxiety disorders, the slight agreement observed in our data ( *κ* =0.17) was somewhat exceeded in the literature with slight-fair agreement ( *κ* =0.29; *κ* ≈0.35).^13^ ^,^^14^ However, while in our sample, agreement was only fair for eating disorders ( *κ* =0.39) and OCD ( *κ* =0.27), in prior meta-analyses, the diagnostic agreement was rather substantial for eating disorders ( *κ* =0.70)^13^ and OCD ( *κ* =0.64).^13^ Finally, we found fair agreement for PTSD ( *κ* =0.40), somewhat lower than the moderate agreement reported for PTSD in previous meta-analyses ( *κ* =0.54).^13^ For antisocial personality disorder, which was not diagnosed in our sample, despite being picked up via the MINI in 5.2%, we were unable to find diagnostic discordance reported by other studies. However, while these differences could be due to study design, patient, and clinician variables mentioned above, importantly, in prior studies, these psychiatric diagnoses were mostly focused on as the primary condition under study, while in our sample, these conditions were comorbid with MDD. These kappa values, ranging from 0.00 to 0.40, indicate slight to fair agreement at best, a level of concordance that is clinically insufficient to guide treatment decisions.

Notably, the diagnostic discordance of comorbidities was primarily due to a substantial underdiagnosis. Specifically, MINI-based comorbid diagnoses were missed in 38% of patients with anxiety disorders, 20% with ASUDs, 15% with PTSD, 7% with OCD, 5% with antisocial personality disorder, and 5% with eating disorders. Reasons for this underdiagnoses could include shame or guilt on the patient’s side, avoidance to ask about sensitive areas on the clinician’s side, time constraints, or diagnostic overshadowing of comorbidities by the main MDD diagnosis. Conversely, psychiatric comorbidities were rarely clinically over-diagnosed, including in 3% with ASUDs, 2% with PTSD, 1% of patients with eating disorders, 1% with OCD, and 0.2% with anxiety disorders. This high rate of underdiagnosis of psychiatric comorbidities may reflect a focus on acute symptoms or index conditions or limited time. Both underdiagnosis and overdiagnosis can result in inappropriate treatment, for example, when PTSD remains unrecognized and only antidepressants are prescribed instead of or without trauma-focused psychotherapy or when subsyndromal substance use is prematurely classified as a full disorder, leading to misdirected clinical attention, stigmatization, and inadequate care.^39^ ^,^^47^

In multivariable regression models examining discordant comorbid diagnoses, the analyses consistently showed that both more MINI-based comorbidities and presence of specific MINI diagnoses were associated with increased likelihood of diagnostic discordance, whereas more clinically recorded comorbidities tended to predict diagnostic concordance. That these two complementary indicators remained consistent predictors across models may point to distinct clinical subgroups: one in which diagnostic complexity is only captured through structured MINIs, and another one in which thorough clinical evaluation more likely results in accurate documentation of comorbidities. An exception to this pattern existed for eating disorders, where more clinically documented comorbidities were associated with increased diagnostic discordance. This finding may indicate that eating disorders are prone to being overlooked even in otherwise detailed clinical assessments, possibly due to their overlapping symptomatology with MDD, particularly regarding appetite and weight changes. Overall, these findings suggest that diagnostic discordance may not primarily reflect ambiguous or poorly defined symptoms but rather systematic differences in the depth of clinical assessments.

In our sample, female sex was frequently associated with diagnostic discordance, though the direction seemed to vary by disorder. Female sex was linked to missed diagnoses of PTSD and eating disorders, conditions underrecognized in our data, likely because more women were at risk for such diagnoses,^48^ ^,^^49^ picked up by the MINI but underrecognized clinically. Although female sex seemed negatively associated with diagnostic discordance in antisocial personality disorder, this was entirely due to being undocumented also by the MINI in females, reflecting lower prevalence and detection rates of antisocial personality disorder in women.^50^ Thus, sex-related variation in diagnostic concordance in our study may be more attributable to sex differences in prevalence, symptom expression, and diagnostic focus than to clinical accuracy per se. Further variables such as adherence, BMI, or illness history were also associated with diagnostic discordance for specific disorders, suggesting additional factors influencing clinical recognition and warrant further investigation.

Taken together, the findings of this study highlight the importance of systematic, structured diagnostic procedures in psychiatry. Although false-positive MDD diagnoses were relatively rare in our sample, they still occurred and often reflected misclassified conditions, such as BD or ASUDs, which can lead to inappropriate treatment with potentially serious consequences. These cases could likely have been avoided through more comprehensive diagnostic assessments. More broadly, the high level of diagnostic discordance in comorbid psychiatric conditions, particularly for antisocial personality disorder and eating disorders, emphasizes the limits of unstructured clinical evaluations. While psychopathological complexity may contribute to such discrepancies, our results suggest that variation in diagnostic practice may also be relevant. In particular, certain subgroups may benefit from targeted structured diagnostic assessment at admission: patients presenting with ASUDs, given their elevated risk of false-positive MDD diagnoses; female patients, given the higher rates of unrecognized PTSD and eating disorders; and patients with a high number of comorbidities, given their consistently higher likelihood of diagnostic discordance. Routine use of brief trauma screeners, such as the Primary Care PTSD Screen, alongside standard admission assessments could, for instance, substantially reduce the rate of missed PTSD diagnoses observed in our sample. Taken together, these findings underline the need for structured and thorough diagnostic procedures to ensure accurate case identification and evidence-based, effective, and cost-conscious treatment allocation. Nevertheless, the time needed to implement measurement-based care is one major obstacle.^51^ Future studies should test the implementation and effectiveness of brief, accurate, and clinical decision-informing assessments in busy treatment settings.

This study has several limitations. First, due to including only patients with a clinical MDD diagnosis, false-negative or true-negative MDD cases cannot be determined. Second, since no false-negative or true-negative MDD cases were available, we could not calculate a kappa coefficients for MDD clinical-based versus research-based diagnostic concordance. However, this design allowed us to focus on the real-world diagnostic accuracy of MDD within inpatient settings, where such diagnoses have immediate clinical consequences. Third, we were unable to conduct a multivariable backward regression analyses including all primary diagnoses that had been missed since BD, substance-related depression, and MDD due to medical condition/substance use were only present among the false-positive MDD cases. Therefore, without these highly relevant determinants of false-positive MDD cases, the multivariable analyses-explained variance was only *r* ^2^=0.08. Nevertheless, the variances explaining misdiagnoses of comorbidities were higher (*r* ^2^=0.21–0.46). Fourth, we were only able to examine comorbidities that were covered by the MINI; thus, other relevant conditions, like attention-deficit/hyperactivity disorder, borderline personality disorder, and autism-spectrum disorders, were not assessed. Fifth, all diagnoses refer to the admission time, and it remains unclear to what extent the diagnostic accuracy may have improved at discharge time. Future studies should compare admission and discharge diagnoses to assess increasing diagnostic accuracy at hospital discharge. However, the diagnoses made upon admission are particularly relevant for early clinical decision-making, underscoring the importance of accurate clinical diagnostic procedures. Sixth, the relatively low annual enrolment per site (~12–13 patients/year) reflects the stringent inclusion criterion of persistent suicidality (≥48 hours postadmission), which restricts the sample to a severe, high-acuity subpopulation rather than indicating selective enrolment. However, this PP2 subgroup was derived from a complete and consecutively admitted sample of 3795 adults hospitalized at the eight study sites^52^—including 2 years of the COVID-19 pandemic with at times reduced inpatient services with MDD. Consecutive enrolment of all eligible patients was mandated by protocol across all eight sites, minimizing the risk of selection bias within this defined population. Nevertheless, findings may not generalize to inpatients with less severe depressive episodes or to nonuniversity inpatient settings. Seventh, residual confounding cannot be excluded, as potentially relevant variables, such as clinician experience, duration of the diagnostic interview, or patient cooperation with the interview and potential symptom concealment, were not systematically captured and may partly account for the observed associations. Such variables should be measured in future studies. Eighth, given the cross-sectional nature of the assessments at admission, directional inferences from the observed associations should be interpreted with caution, as reverse causation cannot be excluded. Finally, differences in clinical or regional settings may limit the generalizability of the findings. Relatedly, inter-rater reliability between hospital staff making diagnostic decision in routine clinical inpatient settings was not assessed. Nonetheless, the multicenter design across eight German psychiatric hospitals provides a diverse sample of routine inpatient care.

In conclusion, this study highlights the need for more standardized, structured, and scalable diagnostic procedures in routine psychiatric care. To improve diagnostic accuracy and ensure that patients receive appropriate and effective treatments, future efforts should aim to integrate structured diagnostic interviews into routine practice, particularly in inpatient settings where patients and clinicians meet several times. Moreover, diagnostic accuracy could be improved via interoperable clinical documentation systems, for instance, through machine learning-based tools aiming to differentiate MDD from BD.^53^ These steps could support a transition toward measurement-based and individualized mental health care, aiming to ultimately improve patient outcomes.

## Long descriptions

### Table 1. Long description

The table consists of six columns: Characteristic, Total n equals 401, Concordant clinical-to-research M D D diagnosis n equals 359, Discordant clinical-to-research M D D diagnosis n equals 42, p-value, and Effect size phi with 95 percent C I.

Key data points include:

* Demographic characteristics: Sex is predominantly female at 57.1 percent total. The p-value for sex is 0.236.

* Family history of psychiatric disorders: 76.2 percent of the total sample, with a p-value of 0.938.

* Socioeconomic status: 45.9 percent are currently employed. Highest educational attainment shows 45.4 percent completed studies or vocational training, with a p-value of 0.408.

* Occupation: The largest group is housekeeper, student, agricultural worker, or unskilled laborer at 25.6 percent. The p-value is 0.088.

* Inpatient admission context: Self-referral is the most common at 52.5 percent. The referral context shows a significant p-value of 0.001.

* Psychiatric emergency: 52.4 percent were in a psychiatric emergency at admission. Suicidality was the primary reason for 49.6 percent of the total sample, with a p-value of 0.856.

* Emergency due to suicidality in the last 24 hours: 7.5 percent total, with 0 percent in the discordant group and a p-value of 0.078.

Navigate back to Table 1..

### Table 2. Long description

The table contains six columns: Clinical characteristics, Total (n = 401), Concordant clinical-to-research M D D diagnosis (n = 359), Discordant clinical-to-research M D D diagnosis (n = 42), p-value, and Effect size phi (95% C I).

Key data sections include:

1. Clinical M D D diagnoses: Severe without psychotic symptoms is the most common (84.3% total). Treatment-resistant depression accounts for 22.9% of the total sample.

2. Comorbidities: Any clinically diagnosed comorbid psychiatric disorder is present in 46.6% of the total. When using the M I N I interview, Bipolar disorder shows a significant difference (p < 0.001) with 0% in the concordant group and 45.2% in the discordant group. Subsyndromal depression also shows a significant difference (p < 0.001) with 21.4% in the discordant group versus 0% in the concordant group.

3. Suicidality: Passive suicidal thoughts are reported by 39.9% of the total. Lifetime suicide attempts are reported by 47.9% of the total sample.

4. Psychotropic medication: 68.3% of the total sample are on medication, with Antidepressants being the most frequent (60.1%). S S R I s (24.4%) and Antipsychotics (26.2%) are also common.

5. Previous treatment: 57.9% have had previous inpatient treatment for depressive episodes, and 81.6% have had lifetime outpatient treatment.

Navigate back to Table 2..

### Table 3. Long description

The table consists of 7 columns: Variable, Total (n = 401), Concordant clinical-to-research M D D diagnosis (n = 359), Discordant clinical-to-research M D D diagnosis (n = 42), p-value, Beta coefficient (beta), and Odds ratio (95% C I).

Key rows include:

* Age, median (I Q R): Total 33.0 (26.0, 47.0); p-value 0.887; Odds ratio 1.00.

* B M I (kg/m super 2), median (I Q R): Total 24.2 (21.4, 28.7); p-value 0.118; Odds ratio 1.05.

* Hollingshead socioeconomic status: Total 37.0 (21.0, 48.0); p-value 0.343; Odds ratio 0.99.

* Number of depressive episodes, mean (S D): Total 3.4 (2.8); p-value 0.408; Odds ratio 0.96.

* Number of comorbid diagnosis, mean (S D): Total 0.7 (1.0); p-value 0.262; Odds ratio 1.23.

* Number of suicide attempts, mean (S D): Total 2.1 (1.8); p-value 0.560; Odds ratio 1.09.

* Number of psychotropic medication, mean (S D): Total 1.4 (1.4); p-value 0.158; Odds ratio 1.21.

* Number of antidepressants, mean (S D): Total 0.8 (0.8); p-value 0.280; Odds ratio 1.27.

* Number of mood stabilizers, mean (S D): Total 0.1 (0.3); p-value 0.710; Odds ratio 1.26.

* Number of antipsychotics, mean (S D): Total 0.30 (0.5); p-value 0.100; Odds ratio 1.92.

All p-values are greater than 0.05, indicating no statistically significant differences between the concordant and discordant groups for these variables.

Navigate back to Table 3..

### Table 4. Long description

The first table displays Primary research-based diagnoses for 401 participants. Columns include M I N I-based diagnoses, Clinical diagnoses, True positive (n = 359), and False negative (n = 42).

* Major depressive disorder: M I N I 359 (89.5%), Clinical 401 (100%), True positive 359 (100%), False negative 0 (0.0%).

* Bipolar disorder: M I N I 19 (4.7%), Clinical 0 (0.0%), False negative 19 (45.2%). This includes Bipolar I (12 cases) and Bipolar II (7 cases).

* Substance use disorder: M I N I 10 (2.5%), Clinical 0 (0.0%), False negative 10 (23.8%).

* Subsyndromal major depressive disorder: M I N I 7 (1.7%), Clinical 0 (0.0%), False negative 7 (16.7%).

* Anxiety disorder: M I N I 4 (1.0%), Clinical 0 (0.0%), False negative 4 (9.5%).

* Secondary depression: M I N I 2 (0.5%), Clinical 0 (0.0%), False negative 2 (4.8%).

The second table displays Research-based comorbid diagnoses for 401 participants. Columns include M I N I-based, Clinical, True positive, False positive, True negative, False negative, Cohen’s Kappa with 95% C I, Standard error, and p-value.

* Posttraumatic stress disorder: M I N I 95 (23.7%), Clinical 43 (10.7%), Kappa 0.40, p < 0.001.

* Eating disorder: M I N I 29 (7.2%), Clinical 14 (3.5%), Kappa 0.39, p = 0.001.

* Substance use disorder: M I N I 114 (28.4%), Clinical 44 (11.0%), Kappa 0.29, p < 0.001.

* Obsessive-compulsive disorder: M I N I 35 (8.7%), Clinical 12 (3.0%), Kappa 0.27, p = 0.031.

* Anxiety disorder: M I N I 181 (45.1%), Clinical 30 (7.5%), Kappa 0.17, p = 0.001.

* Antisocial personality disorder: M I N I 21 (5.2%), Clinical 0 (0.0%), Kappa 0.0.

Navigate back to Table 4..

### Table 5. Long description

The table is divided into two primary sections.

Section 1: False-positive clinical diagnosis of M D D.

Predictors for non-M D D research-based diagnoses include:

* Substance use disorder (M I N I): O R 3.16, p-value 0.001.

* Antidepressant medication: O R 2.97, p-value 0.027.

* Lower number of psychotropic medications: O R 1.67, p-value 0.017.

* Nagelkerke’s R-squared for this model is 0.08.

Section 2: Discordant psychiatric comorbidity in patients with a clinical diagnosis of M D D. This section is broken down by disorder type and level of analysis (number of comorbidities vs. individual comorbidities).

* Anxiety disorders: Higher number of comorbidities (M I N I) is a strong predictor (O R 3.46, p < 0.001). Individual comorbidity predictor is Obsessive-compulsive disorder (O R 2.05, p 0.043). Model R-squared is 0.46 for number and 0.01 for individual.

* Antisocial personality disorder: Absence of antidepressant prescriptions is the highest predictor (O R 10.80, p 0.008). Other predictors include male sex (O R 3.86) and higher number of comorbidities (O R 3.22). Model R-squared is 0.44 for number and 0.18 for individual.

* Posttraumatic stress disorder: Predictors include female sex (O R 2.12, p 0.010) and higher B M I (O R 1.05, p 0.029). Model R-squared is 0.12 for number and 0.07 for individual.

* Eating disorders: Female sex is a major predictor (O R 6.55, p 0.002). Model R-squared is 0.30 for number and 0.08 for individual.

* Obsessive-compulsive disorder: Psychotropic adherence (O R 4.95, p 0.024) and Anxiety disorders (O R 4.10, p 0.001) are key predictors. Model R-squared is 0.23 for number and 0.10 for individual.

* Alcohol and substance use disorders: Predictors include lower number of previous depressive episodes (O R 1.97, p 0.015) and nonadherence (O R 1.88, p 0.032). Model R-squared is 0.21 for number and 0.02 for individual.

Navigate back to Table 5..
